# Cerebellum–Cerebrum paired target magnetic stimulation on balance function and brain network of patients with stroke: A functional near-infrared spectroscopy pilot study

**DOI:** 10.3389/fneur.2022.1071328

**Published:** 2022-12-22

**Authors:** Yifei Xia, Xinwei Tang, Ruiping Hu, Jue Liu, Qun Zhang, Shan Tian, Weining Wang, Ce Li, Yulian Zhu

**Affiliations:** Department of Rehabilitation Medicine, Huashan Hospital, Fudan University, Shanghai, China

**Keywords:** stroke, center of pressure, transcranial magnetic stimulation, cerebellum–cerebrum paired targets, functional near-infrared spectroscopy, functional connectivity

## Abstract

Transcranial magnetic stimulation (TMS) modulation over the cerebellum, primary motor cortex, and supplementary motor cortex individually can improve the balance function of patients with stroke. However, whether their combination could have a better balance modulation effect is uncertain. Therefore, we hypothesized that performing TMS over a combination of these targets can regulate the balance function of patients with stroke. We compared the effects of one-session TMS on eye-open and eye-closed balance conditions in patients with stroke, using different target pairs of unilateral cerebellar (CB-single), cerebellar–primary motor cortex (CB-M1), and cerebellar–supplementary motor area (CB-SMA) targets. A total of 31 patients with stroke were enrolled and randomly divided into three groups to receive single sessions of intermittent theta burst stimulation each. Functional near-infrared spectrum data on resting and standing task states (eye-open and eye-closed) and center of pressure parameters (eye-open and eye-closed) were collected before and after the intervention. Compared with the results in the CB-single group, five intergroup differences in the changes in the center of pressure parameters in the CB-M1 group and two significant differences in the CB-SMA group were observed after one session of intermittent theta burst stimulation. In the CB-SMA group, 12 out of the 14 parameters improved significantly in the EC condition after the intervention. Meanwhile, the functional near-infrared spectrum results showed that the CB-SMA group exhibited a significant inhibitory pattern in the resting-state functional connectivity, which was not observed in the other two groups. In conclusion, we believe that paired targeting of the CB-SMA can reshape the brain network and improve the balance function of patients with stroke.

## Introduction

Stroke is a major reason for motor dysfunction and disability in adults (1, 2). Moreover, approximately half of Chinese patients with intracerebral hemorrhage face death or disability, as reported in 2019. Therefore, the need for motor function rehabilitation has rapidly increased due to the aging population and increasing stroke survivors. The recovery of the balance function is essential to facilitate movement and enable the performance of activities of daily living. Exercise therapy including balance maintaining and weight shifting has been considered a beneficial and commonly used method in improving the balance function of patients with stroke (3). Apart from this, transcranial magnetic stimulation (TMS) is a well-known method for neuroplasticity modulation and has been recommended in stroke rehabilitation (4). Furthermore, many studies reported increased balance and gait stability after TMS modulation sessions (5–11).

The cerebellum is a critical stimulation site in these clinical trials, indicating that it is a promising stimulation target in balance function rehabilitation. Moreover, the neural activity of the cerebral cortex could be adjusted through theta burst stimulation (TBS) over the lateral cerebellum (12). Classically, the improving effect may work in a classical neural circuit, which enables the communication between the cerebellum and cerebrum, called the cerebello-thalamo-cortical (CTC) pathway (13, 14). Some evidence showed that the dentato-thalamo-cortical pathway is the most critical pathway that converts the excitatory information from the cerebellum to more parts of the brain cortex including the primary motor cortex (M1), prefrontal cortex, and supplementary motor area (SMA) (15, 16). M1 is the first choice when it comes to the neural modulation of motor recovery and balance function according to meta-analysis and systematic reviews (10, 11). The reorganization of M1 has been considered a fundamental process in motor rehabilitation. For example, the interactive effect between the cerebellum and M1 has been investigated progressively with easily detectable biological markers using electromyography. Additionally, the SMA plays an important role in balance and gait recovery, wherein it pre-activates in high-demand postural movement that may be a challenge for balance stability, which is reflected by increasing broadband power of theta, alpha, and beta rhythms (17). Based on this fundamental structure, paired targets containing the cerebellum, and cerebral cortex may possibly have more beneficial effects than a single target. Combination targets have been applied to upper limb rehabilitation research in patients with stroke; however, its effects on balance rehabilitation remain unknown (18). Combining SMA or M1 with the cerebellum as stimulation targets seems promising.

Although the cerebellum and some cerebral cortex areas including the SMA and M1 have been proven to be important neural structures in balance function, the neural mechanisms behind this are still uncertain. Considering that the evaluation of the balance function is a dynamic process, functional near-infrared spectroscopy (fNIRS) is the most suitable measurement equipment. Meanwhile, fNIRS has been proven to be sensitive to the changes induced by online and offline TMS protocols (19–21), which makes it an effective way to investigate the neural modulation influence caused by TMS. Previous studies showed that activation changes in the bilateral SMA can be recorded through fNIRS when healthy individuals perform balance tasks after cerebellum-single (CB-single) intermittent TBS (iTBS) (22). The activation of the SMA and dorsolateral prefrontal cortex (DLPFC) is also negatively correlated with the balance function in populations that are healthy or with neurological diseases when balance tasks are highly demanding (23–26). Functional connectivity (FC), as measured through fNIRS, is also a commonly used neural index to describe various brain networks that are characteristic of different diseases (27, 28). This method allows for the exploration of the cortical mechanisms responsible for the recovery of balance function in patients with stroke modulated by TMS, which previous methods could not achieve (26, 29).

Considering all these, we aimed to examine the combinational effect of potential balance-promoting targets. In this pilot study, we set the following target groups: cerebellum-M1 (CB-M1), cerebellum-SMA (CB-SMA), and CB-single. We assume that paired target stimulation works better than single-cerebellum stimulation, wherein paired target groups produce the most significant immediate balance modification effect. Specifically, we suspect that CB-SMA paired targets could improve the balance performance of patients with stroke, as evaluated using the center of pressure (COP). Simultaneously, we expect that FC changes may explain possible neural mechanisms after CB-SMA modulation.

## Methods

### Participants

A total of 31 patients with stroke were included in this randomized, single-blind, parallel-group, pilot study. Outpatients and inpatients were recruited from the Huashan Hospital affiliated with Fudan University, Shanghai, China.

The inclusion criteria are as follows: (1) aged 18–80 years, (2) newly diagnosed with either ischemic or hemorrhagic stroke according to the diagnostic criteria of cerebrovascular diseases in China (version 2019), (3) had a unilateral subacute or chronic stroke caused by the subcortical or cortical lesion (>3 weeks from stroke onset), (4) had motor dysfunction detected using the Fugl–Meyer Assessment of Lower Extremity (FMA-LE, score < 34) and balance dysfunction detected using the Berg Balance Scale (BBS, score < 56), (5) willing to cooperate with evaluations and TMS interventions, and (6) able to stand alone for at least 5 min. The exclusion criteria are as follows: (1) has serious primary diseases of the heart, liver, kidney, and hematopoietic system; (2) has any other non-cerebrovascular diseases that cause limb motor dysfunction; (3) has any metal implants and skull defect; (4) has cancer; and (5) unable to understand or execute commands.

All the patients signed the informed consent and have been informed of possible adverse events before the trial. This study has been approved by the Ethics Committee for Clinical trials of Huashan Hospital affiliated with Fudan University (approval number: 2021-644) and registered with the Chinese Clinical Trial Registry (registration number: ChiCTR2200057240).

### Procedures

**Figure 1:**
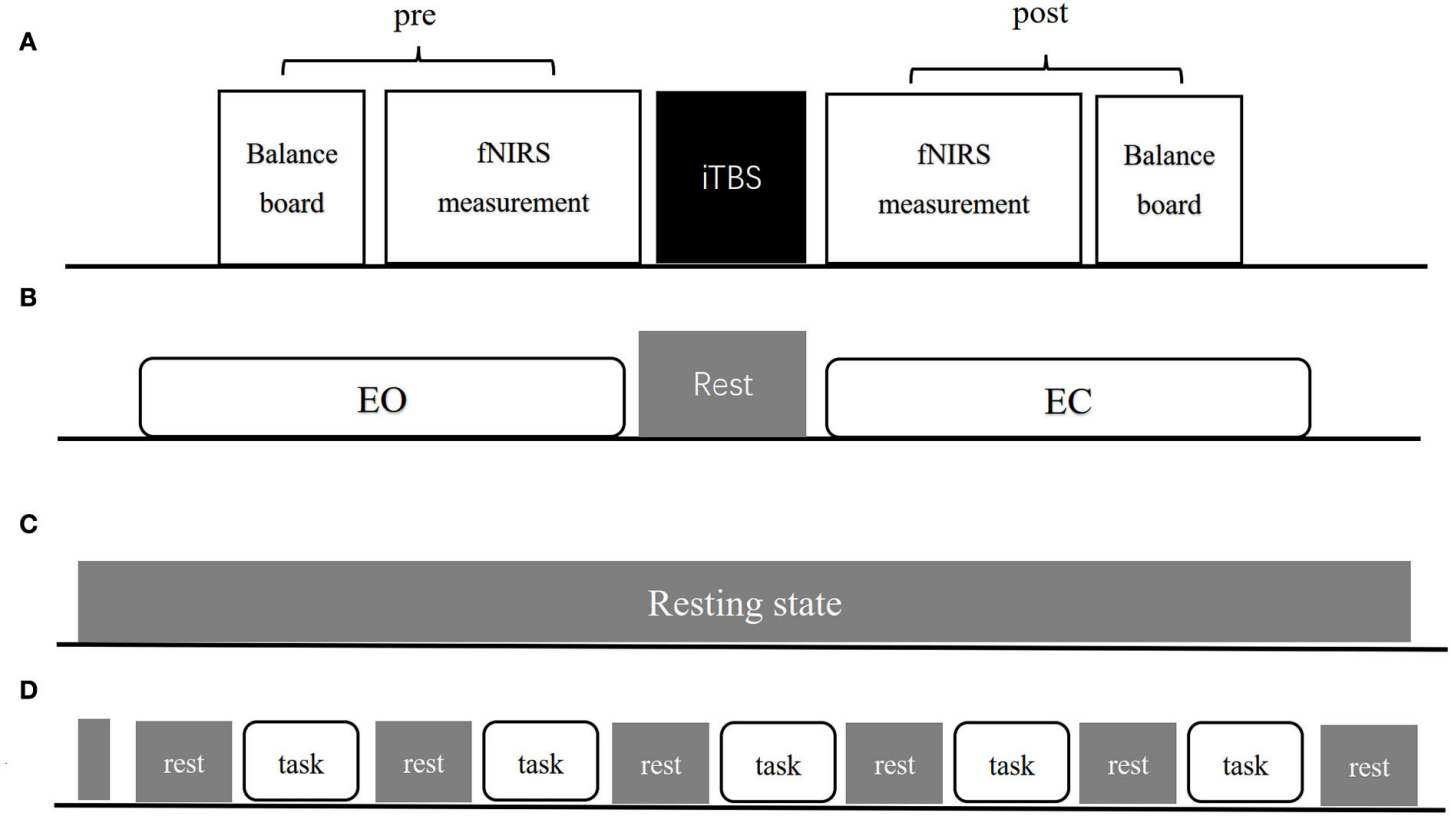
The experimental procedure **(A)** includes pre-measurement, iTBS intervention and post-measurement three parts. **(B–D)** Show exact procedures of pre and post measurement. **(B)** Indicates 1-min EO and EC standing balance measurement separated by 1-min rest. **(C)** Indicates resting-state fNIRS measurement lasting for 8-min. **(D)** Indicates balance task fNIRS measurement organization both in EO and EC condition.

Each patient recruited was first screened using FMA-LE and BBS to identify motor and balance defects. All patients were randomized into three parallel groups—CB-M1, CB-SMA, and CB-single—using a computer-generated randomization list according to a therapist who was not involved in this study, in which patients were not clear about which group they were in. Subsequently, all patients followed the experimental procedures presented in Figure 1, including balance system evaluation, fNIRS measurement, and TMS intervention. Another round of balance and fNIRS measurement was conducted immediately after the TMS intervention.

### Balance system

Center of pressure parameters including “COP speed (COP_*s*_),” “Acceleration of COP (COP_*a*_),” “medial-lateral (ML)-dispersion of COP (ML-COP_*d*_),” “anterior-posterior (AP)-dispersion of COP (AP-COP_*d*_),” “ML-speed of COP (ML-COP_*s*_),” “AP-speed of COP (AP-COP_*s*_),” “ML-acceleration of COP (ML-COP_*a*_),” “AP-acceleration of COP (AP-COP_*a*_),” “Long-time dispersion degree of COP movement area (L-COP_*da*_),” “Short-time dispersion degree of COP movement area (S-COP_*da*_),” “Long-time dispersion of COP velocity (L-COP_*dv*_),” “Short-time dispersion of COP velocity (S-COP_*dv*_),” “COP movement area (COP_*area*_),” and “Score” were used to describe the balance function of every patient. We set the “Score” as the primary outcome. All the parameters were collected using BalanceMotus^TM^ (FP-A-1, Fourier intelligence), which contains a force platform, three armrests, and a computer system. Patients were asked to perform a 1-min balance evaluation, in which they were instructed to stand on the platform with two arms down naturally, under two conditions—eyes open (EO) and closed (EC)—separately. One therapist operated the system and stood aside to ensure the safety of the patients. Evaluation time points before and after the TMS intervention were included.

### Functional near-infrared spectroscopy

**Figure 2:**
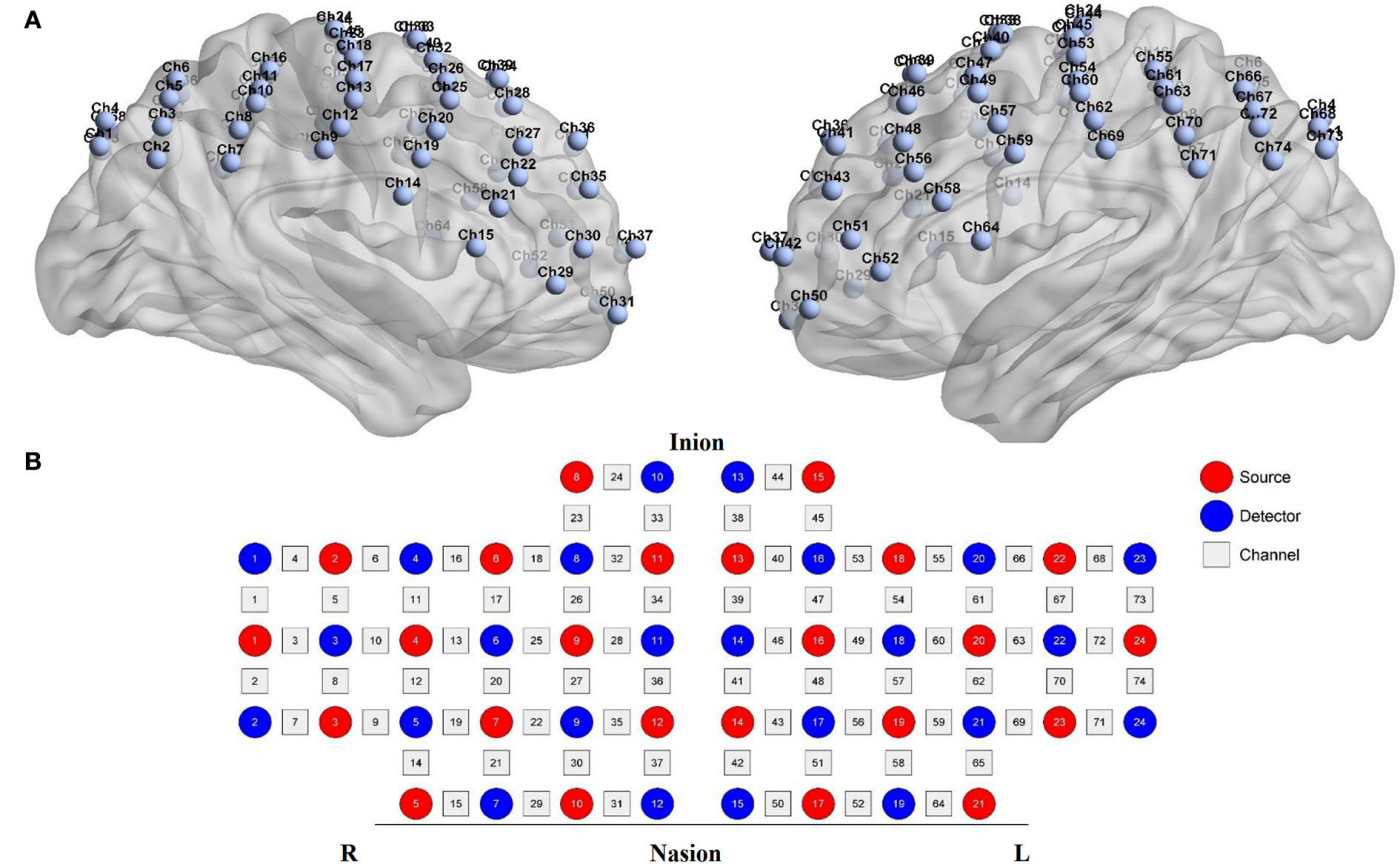
fNIRS montage. **(A)** Channels arrangements with numbers marked on a three-dimensional brain model (ICBM 152 Non-linear Atlases, version 2019) in left and right vision. **(B)** Graphic design of each source, detector, and channel.

Functional near-infrared spectroscopy data were acquired using a 74-multichannel fNIRS instrument device (NirScan, Danyang Huichuang Medical Equipment Co. Ltd.) with a sampling rate of 11 Hz. The wavelengths were set at 730 and 850 nm. Hemodynamic responses were recorded from the resting state and two block-design balance tasks (EO and EC). The source and detector probe montage and cortical representation area are shown in Figure 2 and Table 1. The majority of the prefrontal, partial parietal, and occipital lobes were covered. The distance between the sources and detectors was 3 cm. fNIRS recording was performed by one well-trained therapist in a specific therapy room with absorbing cotton insulation around the wall. Additionally, the light was also turned off to avoid any influence on the recording. The fNIRS measurement was conducted before and immediately after the TMS session.

**Table 1:** The brain representative area with a most percentage on a Brodmann template under each channel (74 channels).

**ROI**	**Region**	**Channels—right hemisphere**	**Channels—left hemisphere**
V3	19-V3	1, 4	68, 73
Broca network	45—pars triangularis Broca's area	15, 21, 22	52,56
	44—pars opercularis, part of Broca's area		58
Wernicke network	39—Angular gyrus, part of Wernicke's area	2, 3, 5	66, 67, 72, 74
	40—Supramarginal gyrus part of Wernicke's area	7, 8, 10, 11	61, 63, 70, 71
Sensory network	1, 2, 3—Primary somatosensory cortex	9, 12, 16	55, 60, 62, 69
	7—Somatosensory association cortex	6	
Motor network	6—Pre-Motor and supplementary motor cortex	19, 20, 23, 24, 33	38, 40, 44, 45, 47, 57, 59, 64
	4—Primary motor cortex	13, 17, 18	53, 54
DLPFC network	9—Dorsolateral prefrontal cortex	25, 26, 28, 36	41, 46, 49
	9, 46—Dorsolateral prefrontal cortex	27	48
	46—Dorsolateral prefrontal cortex	29, 30, 35	43, 51, 50
Prefrontal	10—Frontopolar area	37	42
	8—Includes frontal eye fields	32, 34	39

The resting-state measurement lasted for 8 min to acquire stable hemodynamic information. Patients were asked to open their eyes, keep their heads stable, and avoid falling asleep and any deliberate head movement for the whole period. A block design was used under both EO and EC balance conditions. Every block contains a 30 s rest time and a 35 s balance task. One balance measurement consisted of 10 s of baseline measurement, five blocks, and another 30 s of rest. Every patient was asked to sit on an armchair initially and keep their body still with their eyes open when they hear the command, “keep rest.” After hearing the command, “keep balance,” the patient was instructed to stand up with (or without) necessary help from the therapist and then keep their body balance under EO or EC condition for 30 s. The patients were allowed to sit down to avoid fatigue when they hear the “keep rest” command again. They were instructed to refrain from conversations and additional head movement during the balance-keeping process. The therapist stood beside the patients for safety.

### Transcranial magnetic stimulation

A figure-of-eight cooled coil (YRD204F, diameter of 90 mm) was used to perform the magnetic stimulation in both resting motor threshold evaluation and iTBS intervention protocol (NS 5000, YIRUIDE, Wuhan, China).

Before iTBS interventions, the patients were asked to sit comfortably in the armchair while accepting single TMS to the primary motor cortex ipsilateral to the affected hemisphere to identify the resting motor threshold. The resting motor threshold was defined as the minimal stimulation intensity which could evoke at least five out of 10 times motor evoked potentials peak-to-peak wave amplitude larger than 50 μV in the first dorsal interosseous muscle. Meanwhile, the stimulation site was defined as the motor hot spot of the hemisphere. The motor-evoked potential amplitudes were recorded using surface electromyography with a pair of Ag-Ag/Cl electrodes. The hot spot on the contralateral hemisphere was also marked and converted to the mirror site in the absence of an ipsilateral motor hot spot.

One session of iTBS protocol consisted of bursts containing three pulses at 50 Hz repeated at 5 Hz, in which a total of 600 pulses were delivered in 192 s. The iTBS stimulation sites contained M1, SMA, and CB which were figured out through motor hot spots and bony landmarks. Specifically, the motor hot spot was considered the M1 stimulation site. The SMA was defined as 3 cm in front of Cz and 0.5 cm close to the hemisphere (30, 31). The cerebellum stimulation point was defined as 1 cm below and 3 cm lateral to the inion (7). All patients allocated to the CB-M1 group received iTBS intervention on the cerebellum hemisphere contralateral to the affected cerebrum hemisphere and M1 ipsilateral to the affected side. The same protocol was performed in the CB-SMA group, except that the M1 site was converted to the SMA site. The two iTBS sessions were delivered to the cerebellum first then the cerebrum continuously without time break by an experienced TMS therapist, as well as adverse events recording. The patients allocated in the CB-single group received a one-cerebellum iTBS session.

### fNIRS data processing

The NIRS-KIT (32) toolbox is a MATLAB-based fNIRS analysis package designed by Beijing Normal University, which contains resting-state and task fNIRS analysis functions including the following steps: data conversion, previewing, processing, individual-level analysis, group-level analysis, and visualization. MATLAB version R2013b (MathWorks, USA) was used to operate NIRS-KIT and other classical fNIRS analysis toolboxes including Homer2 (33) and SPM8 (34). In addition, BrainNet Viewer (35) was used for 3D visualization in addition to the 2D pictures drawn by NIRS-KIT. Space registration was performed according to the NFRI method to obtain Montreal Neurological Institute coordinates (36).

Pre-processing was applied to both task-related and resting-state fNIRS data following the steps later. First, the original light intensity was converted to optical density. Second, a customized script was applied to realize the data mirror conversion between the converting and processing steps. As a result, the right brain hemisphere was considered as the affected side, as well as the intervened cerebral hemisphere, among all the patients in the fNIRS data analysis, which was previously practiced (26, 37, 38). Third, each data term was previewed in the data viewer window to check for the quality of raw data and determine the pre-processing parameters. We excluded one patient in the CB-M1 group because of the low data quality. Fourth, a polynomial regression model was used to realize detrending. The temporal derivative distribution repair method (39) was applied to motion correction. A third-order Butterworth filter (infinite impulse response) with a cutoff frequency of 0.01–0.08 Hz was operated to remove the noise signals (heart rate, breath, and other low-frequency signal drift) that are not of interest. Optical density signals were converted to blood oxygen data according to the modified Beer–Lambert law including the oxygenated hemoglobin, deoxyhemoglobin, and total hemoglobin data. Oxygenated hemoglobin was chosen for further analysis in the next individual analysis step due to its superior sensitivity.

### FC analysis

Functional connectivity was calculated using oxygenated hemoglobin through two methods: FC matrix analysis and region of interest (ROI) to whole-brain FC. Pearson's correlation coefficient was selected for calculation, as well as the Fisher-Z score convert, which was completed in the individual analysis. Subsequently, group analysis was conducted in each group in comparison with pre- and post-iTBS. False discovery rate (FDR) was used to correct the *P*-values (α = 0.05) since multiple comparisons were performed in 74 channels.

#### Whole-brain FC matrix

The whole-brain FC matrix was used to calculate functional connectivity through all channel pairs, which represented connectivity strength. The Fisher-score matrix was set at a density under a threshold of *P* = 0.05 for visualization. We also performed a subnet mark to highlight important functional representative areas in the brain. For 3D visualization, channels were defined as nodes and connectivity values as edges, where quite outstanding edges would be displayed in the 3D brain map.

The 74 channels were divided into 14 subnets for better understanding according to the Brodmann area. The details of the right hemisphere network are as follows: V3 (Ch. 1, 4), Broca's network (Ch. 15, 21, 22), Wernicke network (Ch. 2, 3, 5, 7, 8, 10, 11), sensory network (Ch. 6, 9,12, 16), motor network (Ch. 13, 17, 18, 19, 20, 23, 24, 33), DLPFC network (Ch. 25, 26, 27, 28, 29, 30, 35, 36), and prefrontal (Ch. 31, 32, 34, 37). The details of the left hemisphere are as follows: V3 (Ch. 68, 73), Broca's network (Ch. 52, 56, 58), Wernicke network (Ch. 61, 63, 66, 67, 70, 71, 72, 74), sensory network (Ch. 55, 60, 62, 69), motor network (64, 59, 57, 54, 47, 53, 45, 44, 38, 40), DLPFC network (Ch. 41, 43, 46, 48, 49, 51), and prefrontal network (Ch. 39, 42, 50).

#### ROI to whole-brain

Previous research demonstrated that motor function rehabilitation is definitely beneficial for balance recovery, which may be due to the centralities increased within the ipsilateral M1. In this case, an ROI, which included the cortical representative areas of M1, SMA, and premotor cortex (PMC) and also located on the stimulated hemisphere, was chosen to investigate the modulation effect of NC-MS pairs on FC from ROI to the other brain areas. The ROI contains channels 13, 17, 18, 19, 20, 23, 24, and 33.

### Task-related cortical activation analysis

The analysis of neural activation in task-related condition estimation was performed on the basis of the general linear model after data processing. On a first-level analysis, the brain activity undergoing the balance task was analyzed, focusing on the contrast between the EC/EO and resting conditions. For the block-design balance task condition, the time ranged from 5 to 35 s after the stimulation onset to avoid hemodynamic interference caused by body motion (40). In contrast, a 30 s resting period was fully taken into account. Task-related signal changes were calculated as a beta value for further analysis after finishing the contrast. Subsequently, group-level analysis was performed on beta values. Significance was set at *P* < 0.05 (uncorrected).

### Other statistical analyses

IBM SPSS Statistics for Windows, version 25 (IBM Corp., Armonk, N.Y., USA) was utilized for statistical analysis with the threshold for significance set at *P* < 0.05. The assumption of normality for variables was assessed using the Shapiro–Wilk test. All data are presented as mean ± SD, *N* (%), or mid (interquartile range). The baseline differences between the three groups were detected using a one-way analysis of variance (age, FMA-LE, and BBS), chi-square test (gender, primary diagnosis, and paralysis side), and Kruskal–Wallis test (education years and course of stroke). The Kruskal–Wallis test was used with group factors (CB-SMA, CB-M1, and CB-single) to compare the change from baseline scores (post–pre: Δvalue) between the groups. The paired sample *t*-test and Wilcoxon test were chosen to detect within-group differences between pre- and post-iTBS intervention according to the homogeneity of variance and normality of the COP data.

## Results

**Figure 3:**
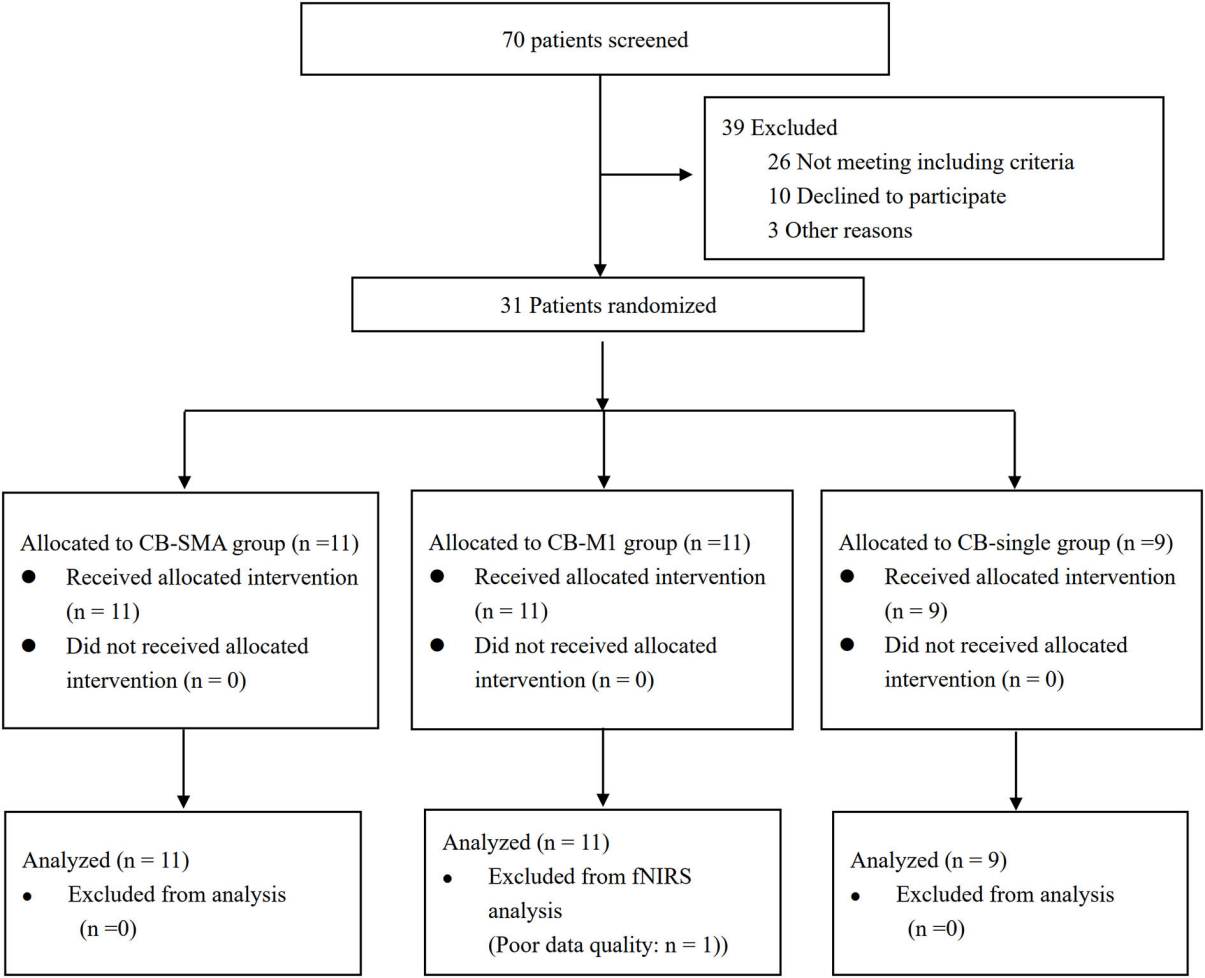
CONSORT flow diagram of patients enrolled in the study.

The Consolidated Standards of Reporting Trials (CONSORT) patients' flowchart is shown in Figure 3. The clinical characteristics of the three groups are shown in Table 2. Differences were not observed in the patient's demographic characteristics including age, gender, course of a stroke, FMA-LE, BBS, primary diagnosis, and paralysis side among the three groups in the baseline. All patients completed the intervention and measurement process, and no adverse events happened in all groups.

**Table 2:** Baseline characteristics of the three patients' groups.

	**CB-SMA (*n* = 11)**	**CB-M1 (*n* = 11)**	**CB-single (*n* = 9)**	***P-*value**
Age (years)	50.36 ± 8.99	47.18 ± 11.98	54.44 ± 14.21	0.400^a^
Gender (male/female)	10/1	10/1	8/1	0.985^b^
Course of stroke (days)	50.00 (13.00)	50.00 (17.00)	49.00 (25.00)	0.861^c^
FMA-LE	23.45 ± 4.72	22.64 ± 6.76	23.44 ± 5.08	0.929^a^
BBS	43.00 ± 11.58	35.45 ± 12.08	41.56 ± 12.25	0.311^a^
Education (years)	12.00 ± 7.00	16 ± 7.00	12.00 ± 9.00	0.708^c^
Primary diagnosis				0.878^b^
Hemorrhagic	5 (45.6%)	6 (54.5%)	4 (44.4%)	
Ischemic	6 (54.5%)	5 (45.5%)	5 (55.6%)	
Paralysis side				0.878^b^
Right	5 (45.5%)	6 (54.5%)	5 (55.6%)	
Left	6 (54.5%)	5 (45.5%)	4 (44.4%)	

Data are expressed as Mean ± SD, N (%), or Mid (IQR).

^a^One-way ANOVA;

^b^Chi–square test;

^c^Kruskal–Wallis test.

### COP evaluation

All the parameters have been tested according to their normality and homogeneity of variance. Significant differences in the EO condition were observed among the three groups in the following parameters (Table 3): ΔCOP_*a*_ (*P* = 0.028), ΔAP-COP_*d*_ (*P* = 0.048), ΔAP-COP_*s*_ (*P* = 0.011), ΔAP-COP_*a*_ (*P* = 0.025), and ΔS-COP_*dv*_ (*P* = 0.012). Subsequently, *post-hoc* analysis was performed using Bonferroni correction. In ΔAP-COP_*d*_, the CB-SMA target group showed a great decline after magnetic stimulation than the CB-M1 group (*P* = 0.043). In ΔAP-COP_*s*_, the CB-M1 (*P* = 0.022) and CB-SMA (*P* = 0.027) target groups showed a significant decline compared to the CB-single target group. The CB-M1 target group showed a significant decline to the CB-single target group in ΔAP-COP_*a*_ (*P* = 0.034), ΔS-COP_*dv*_ (*P* = 0.012), and ΔCOP_*a*_ (*P* = 0.038) values. Additionally, we found that AP-COP_*d*_ (*P* = 0.028) and ML-COP_*a*_ (*P* = 0.048) improved significantly in the CB-SMA group, as well as ML-COP_*a*_ (*P* = 0.024) improvement in the CB-M1 target group. No significant improvement was observed after CB-single stimulation.

**Table 3:** Balance board parameters in eyes open condition.

		**CB-SMA (*n* = 11)**	**CB-M1 (*n* = 11)**	**CB-single (*n* = 9)**	***P*-between (Δvalue)**
COP*_*s*_*	Pre	15.12 ± 1.82	16.44 ± 2.76	12.88 ± 1.81	0.153
	Post	13.08 ± 1.80	13.34 ± 2.05	12.91 ± 2.01	
	*P*-within	0.102	0.067	0.972	
COP*_*a*_*	Pre	157.4 ± 19.36	178.78 ± 31.22	137.49 ± 18.39	0.028^c^^*^
	Post	138.48 ± 19.27	143.85 ± 21.68	144.51 ± 18.54	
	*P*-within	0.075	0.128	0.315	
ML-COP*_*d*_*	Pre	6.74 ± 1.72	7.06 ± 1.53	5.09 ± 0.96	0.137
	Post	7.56 ± 1.70	5.34 ± 1.00	5.31 ± 1.18	
	*P*-within	0.439	0.091	0.859	
AP-COP*_*d*_*	Pre	6.81 ± 0.79	5.41 ± 0.47	5.17 ± 0.59	0.048^c^^*^
	Post	5.75 ± 0.64	5.59 ± 0.52	5.05 ± 0.82	
	*P*-within	0.028^a^^*^	0.587	0.752	
ML-COP*_*s*_*	Pre	7.61 ± 1.13	8.23 ± 1.79	7.05 ± 1.36	0.906
	Post	6.16 ± 1.04	6.31 ± 1.22	6.15 ± 1.35	
	*P*-within	0.154	0.057	0.196	
AP-COP*_*s*_*	Pre	11.25 ± 1.38	12.11 ± 1.87	9.18 ± 1.08	0.011^c^^*^
	Post	10.00 ± 1.26	10.14 ± 1.45	9.85 ± 1.20	
	*P*-within	0.060	0.126	0.059	
ML-COP*_*a*_*	Pre	68.93 ± 9.04	71.64 ± 11.57	62.90 ± 10.52	0.056
	Post	54.05 ± 8.72	56.67 ± 8.43	62.15 ± 10.44	
	*P*-within	0.048^a^^*^	0.024^b^^*^	0.859	
AP-COP*_*a*_*	Pre	126.29 ± 16.34	145.81 ± 26.90	108.13 ± 12.07	0.025^c^^*^
	Post	114.38 ± 15.28	119.23 ± 18.16	117.58 ± 12.70	
	*P*-within	0.062	0.196	0.154	
L-COP*_*da*_*	Pre	6.94 ± 1.08	6.17 ± 1.02	5.21 ± 0.76	0.579
	Post	7.00 ± 1.27	5.61 ± 0.65	4.80 ± 0.84	
	*P*-within	0.935	0.393	0.382	
S-COP*_*da*_*	Pre	0.10 ± 0.01	0.09 ± 0.02	0.08 ± 0.01	0.199
	Post	0.08 ± 0.01	0.08 ± 0.01	0.08 ± 0.01	
	*P*-within	0.071	0.657	1.000	
L-COP*_*dv*_*	Pre	14.95 ± 1.98	15.76 ± 2.66	12.40 ± 1.99	0.245
	Post	13.14 ± 2.02	13.28 ± 2.04	12.94 ± 2.10	
	*P*-within	0.238	0.082	0.462	
S-COP*_*dv*_*	Pre	1.13 ± 0.14	1.27 ± 0.23	0.97 ± 0.13	0.012^c^^*^
	Post	1.00 ± 0.15	1.01 ± 0.15	1.04 ± 0.14	
	*P*-within	0.135	0.120	0.141	
COP*_*a*_*	Pre	948.15 ± 326.65	828.92 ± 243.31	576.00 ± 230.03	0.730
	Post	759.84 ± 172.07	687.39 ± 199.13	576.77 ± 232.63	
	*P*-within	0.502	0.275	0.989	
Score	Pre	58.27 ± 9.40	53.36 ± 9.69	46.67 ± 10.71	0.401
	Post	52.09 ± 8.82	46.55 ± 10.18	46.89 ± 10.68	
	*P*-within	0.169	0.103	0.967	

COP_*s*_, COP Speed; COP_*a*_, Acceleration of COP; ML-COP_*d*_, ML-dispersion of COP; AP-COP_*d*_, AP-dispersion of COP; ML-COP_*s*_, ML-speed of COP; AP-COP_*s*_, AP-speed of COP; ML-COP_*a*_, ML-acceleration of COP; AP-COP_*a*_, AP-acceleration of COP; L-COP_*da*_, Long-time dispersion degree of COP movement area; S-COP_*da*_, Short-time dispersion degree of COP movement area; L-COP_*dv*_, Long time dispersion of COP velocity; S-COP_*dv*_, Short time dispersion of COP velocity; COP_*a*_, COP movement area. Δvalue, the difference value in post and pre;

^a^Paired-t test;

^b^Wilcoxon;

^c^Kruskal–Wallis test;

^*^P < 0.05.

In the EC condition, only L-COP_*da*_ (*P* = 0.085) showed a trend of difference among the three groups (Table 4). However, a remarkable improvement was observed after iTBS intervention was applied to the CB-SMA targets. Here, COP_*s*_, COP_*a*_, ML-COP_*a*_, S-COP_*da*_, and L-COP_*dv*_ descend significantly (*P* < 0.01), as well as ML-COP_*d*_, AP-COP_*d*_, ML-COP_*s*_, L-COP_*da*_, S-COP_*dv*_, COP_*area*_, and score (*P* < 0.05). Only S-COP_*da*_ (*P* = 0.026) declined after stimulation on the CB-M1 brain targets. No significant changes appeared in the CB-single group.

**Table 4:** Balance board parameters in eyes closed condition.

		**CB-SMA (*n* = 11)**	**CB-M1 (*n* = 11)**	**CB-single (*n* = 9)**	***P*-between (Δvalue)**
COP*_*s*_*	Pre	25.59 ± 3.19	27.78 ± 3.25	23.34 ± 2.70	0.935
	Post	22.45 ± 2.72	24.07 ± 3.56	20.16 ± 2.18	
	*P*-within	0.005^a^^**^	0.081	0.106	
COP*_*a*_*	Pre	279.77 ± 42.00	302.19 ± 40.19	254.03 ± 28.38	0.765
	Post	241.38 ± 36.54	257.34 ± 40.63	233.31 ± 20.52	
	*P*-within	0.006^a^^**^	0.053	0.258	
ML-COP*_*d*_*	Pre	9.15 ± 1.67	7.83 ± 1.14	7.91 ± 1.79	0.323
	Post	6.46 ± 0.84	7.91 ± 1.25	7.26 ± 1.44	
	*P*-within	0.040^a^^*^	0.912	0.492	
AP-COP*_*d*_*	Pre	9.21 ± 0.80	8.86 ± 0.79	8.40 ± 1.07	0.509
	Post	7.37 ± 0.58	8.26 ± 0.72	7.18 ± 0.85	
	*P*-within	0.010^a^^*^	0.335	0.296	
ML-COP*_*s*_*	Pre	11.50 ± 1.85	12.95 ± 2.06	10.57 ± 1.58	0.726
	Post	9.09 ± 1.20	11.70 ± 2.45	8.73 ± 1.57	
	*P*-within	0.019^b^^*^	0.280	0.126	
AP-COP*_*s*_*	Pre	19.97 ± 2.37	21.20 ± 2.54	18.32 ± 1.86	0.733
	Post	18.52 ± 2.33	18.22 ± 2.32	16.09 ± 1.60	
	*P*-within	0.079	0.078	0.150	
ML-COP*_*a*_*	Pre	105.29 ± 17.08	115.35 ± 14.39	97.91 ± 13.00	0.445
	Post	84.25 ± 13.53	102.21 ± 19.28	90.15 ± 13.48	
	*P*-within	0.004^a^^**^	0.167	0.384	
AP-COP*_*a*_*	Pre	235.37 ± 36.93	149.24 ± 35.91	213.76 ± 23.88	0.962
	Post	208.61 ± 32.39	212.81 ± 32.31	196.88 ± 17.16	
	*P*-within	0.053	0.079	0.310	
L-COP*_*da*_*	Pre	9.19 ± 1.09	8.10 ± 0.85	7.51 ± 0.90	0.085
	Post	6.91 ± 0.53	8.04 ± 0.96	6.64 ± 0.80	
	*P*-within	0.012^a^^*^	0.905	0.313	
S-COP*_*da*_*	Pre	0.16 ± 0.02	0.18 ± 0.02	0.14 ± 0.02	0.837
	Post	0.14 ± 0.02	0.15 ± 0.02	0.12 ± 0.01	
	*P*-within	0.004^a^^**^	0.026^a^^*^	0.078	
L-COP*_*dv*_*	Pre	26.08 ± 3.18	27.35 ± 3.60	22.86 ± 2.62	0.710
	Post	22.24 ± 2.64	22.96 ± 3.45	20.12 ± 2.24	
	*P*-within	0.002^a^^**^	0.051	0.122	
S-COP*_*dv*_*	Pre	2.07 ± 0.32	2.16 ± 0.31	1.85 ± 0.19	0.820
	Post	1.80 ± 0.28	1.82 ± 0.28	1.69 ± 0.14	
	*P*-within	0.024^a^^*^	0.055	0.245	
COP*_*a*_*	Pre	1,742.01 ± 331.13	1,423.02 ± 425.99	1,529.57 ± 493.14	0.142
	Post	1,031.92 ± 150.10	1,417.93 ± 368.54	961.55 ± 285.25	
	*P*-within	0.014^a^^*^	0.978	0.084	
Score	Pre	85.45 ± 4.36	84.27 ± 5.20	78.78 ± 7.42	0.594
	Post	76.55 ± 6.58	78.00 ± 8.17	76.33 ± 7.66	
	*P*-within	0.001^b^^*^	0.287	0.704	

COP_*s*_, COP Speed; COP_*a*_, Acceleration of COP; ML-COP_*d*_, ML-dispersion of COP; AP-COP_*d*_, AP-dispersion of COP; ML-COP_*s*_, ML-speed of COP; AP-COP_*s*_, AP-speed of COP; ML-COP_*a*_, ML-acceleration of COP; AP-COP_*a*_, AP-acceleration of COP; L-COP_*da*_, Long-time dispersion degree of COP movement area; S-COP_*da*_, Short-time dispersion degree of COP movement area; L-COP_*dv*_, Long time dispersion of COP velocity; S-COP_*dv*_, Short time dispersion of COP velocity;COP_*a*_, COP movement area. Δvalue, the difference value in post and pre;

^a^Paired-t test;

^b^Wilcoxon; ^c^Kruskal–Wallis test;

^*^P < 0.05;

^**^P < 0.01.

### Resting-state FC

Resting-state data were compared between the post- and pre-iTBS sessions. A completely different FC model has been observed among the CB-M1, CB-SMA, and CB-single targets. Specifically, the CB-SMA group exhibited an overall inhibitive model, while a relatively unchanged connectivity model with limited excitation was observed in the CB-M1 group after the TMS intervention. However, non-important network connection modification has been witnessed in the CB-single target group.

#### Whole-brain FC matrix

**Figure 4:**
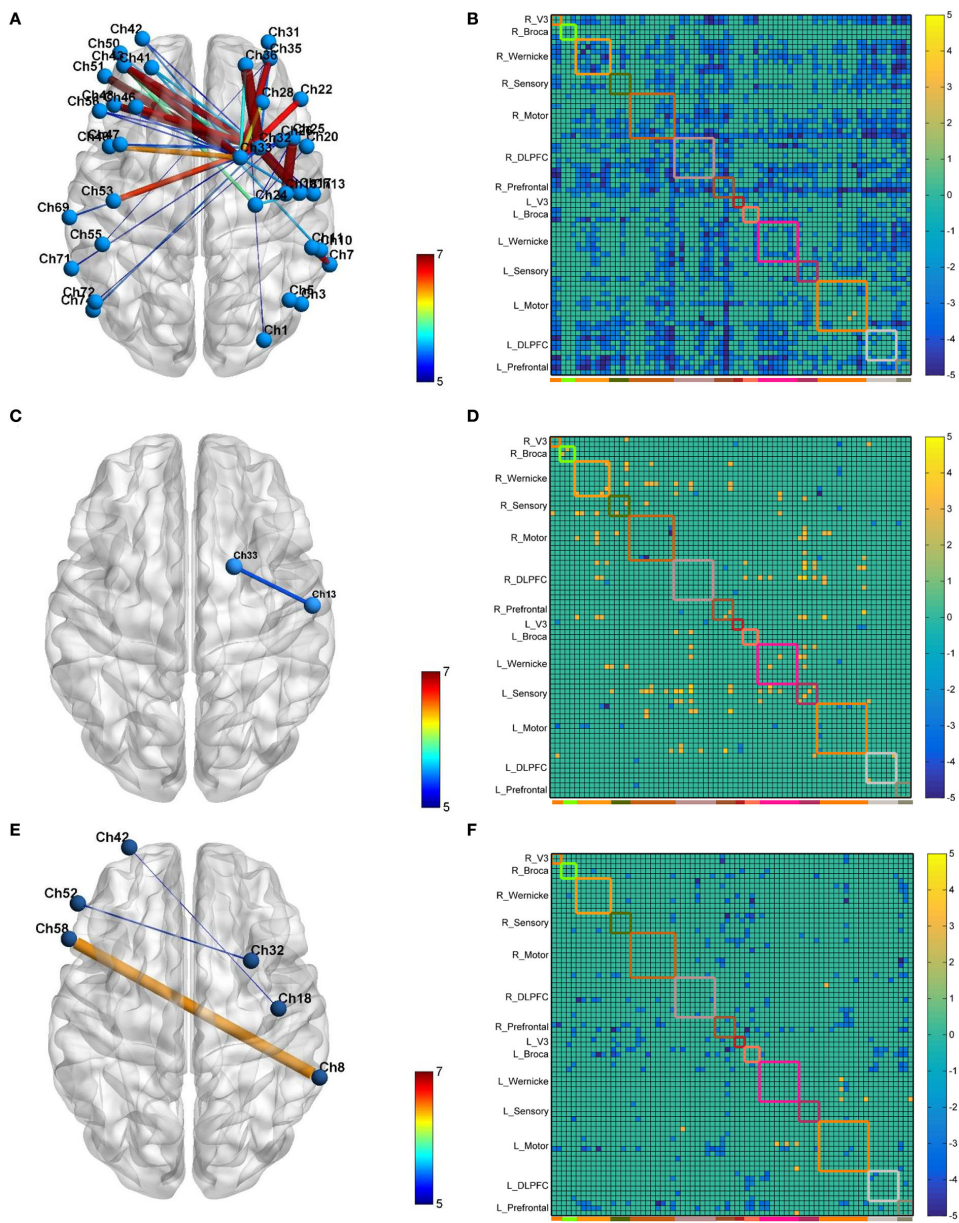
The 3D brain visualization of the difference in the connectivity after CB-SMA **(A)** protocol, CB-M1 protocol **(C)**, and CB-single **(E)**. The FC Matrix of different connections between post- and pre-iTBS intervention in CB-SMA group **(B)**, CB-M1 group **(D)** and CB-single **(F)**. Significant connections *(p* < 0.05, FDR corrected) after paired *t*-test are colored contrast to background color according to *T*-values. Subnets and within connections are highlighted by rectangle.

The neural modification effect of the three iTBS protocols is quite different, as reflected by the FC matrix (Figure 4). A sparsity threshold of 0.01 on edge was set to visualize the 3D mapping (Figure 4) of the strongest FC connections. The overall nature of the CB-SMA target group was an inhibition model in contrast to the relatively unchanged models presented by the CB-M1 and CB-single target groups.

In the CB-SMA target group, the channels that were able to meet the FDR-corrected p threshold (*P* < 0.0046) are highlighted with a color bar in the FC matrix and 3D brain (Figure 4A). The most significant reduction in connectivity between Ch. 7 and Ch. 11 (*T* = −7,746, *P* < 0.001) was observed in the affected hemisphere. Inter-hemisphere decrease was mainly in the connecting right side of the SMA and DLPFC, containing channels 33, 32, and 26, with contralateral PMC (Ch. 53, *T* = −6.678, *P* < 0.001) and DLPFC (Ch. 43, *T* = −6.576, *P* < 0.001). Additionally, within-hemisphere FC changes in the right hemisphere were featured on Ch. 36–32 (*T* = −6.115, *P* < 0.001) and Ch. 36–1 (*T* = −6.084, *P* < 0.001).

In the CB-M1 target group, only one connection (Figure 4C) between Ch. 33 and 13 survived, representing a decreased effect within the motor subnets. Some increased connectivity was observed between different subnets (R_Wernicke, L_Wernicke, and L_Sensory) in the FC matrix, which is quite different from the matrix of the CB-SMA group. In the CB-single target group, three connections from Ch. 32, 18, and 8 in the right brain area (R_Prefrontal, R_Motor, and R_Wernicke) to the Ch. 52, 42, and 58 in the left brain (L_Broca and L_Prefrontal) were observed. However, none of these changes in these two groups remained significantly different after the FDR correction.

#### ROI to whole brain

**Figure 5:**
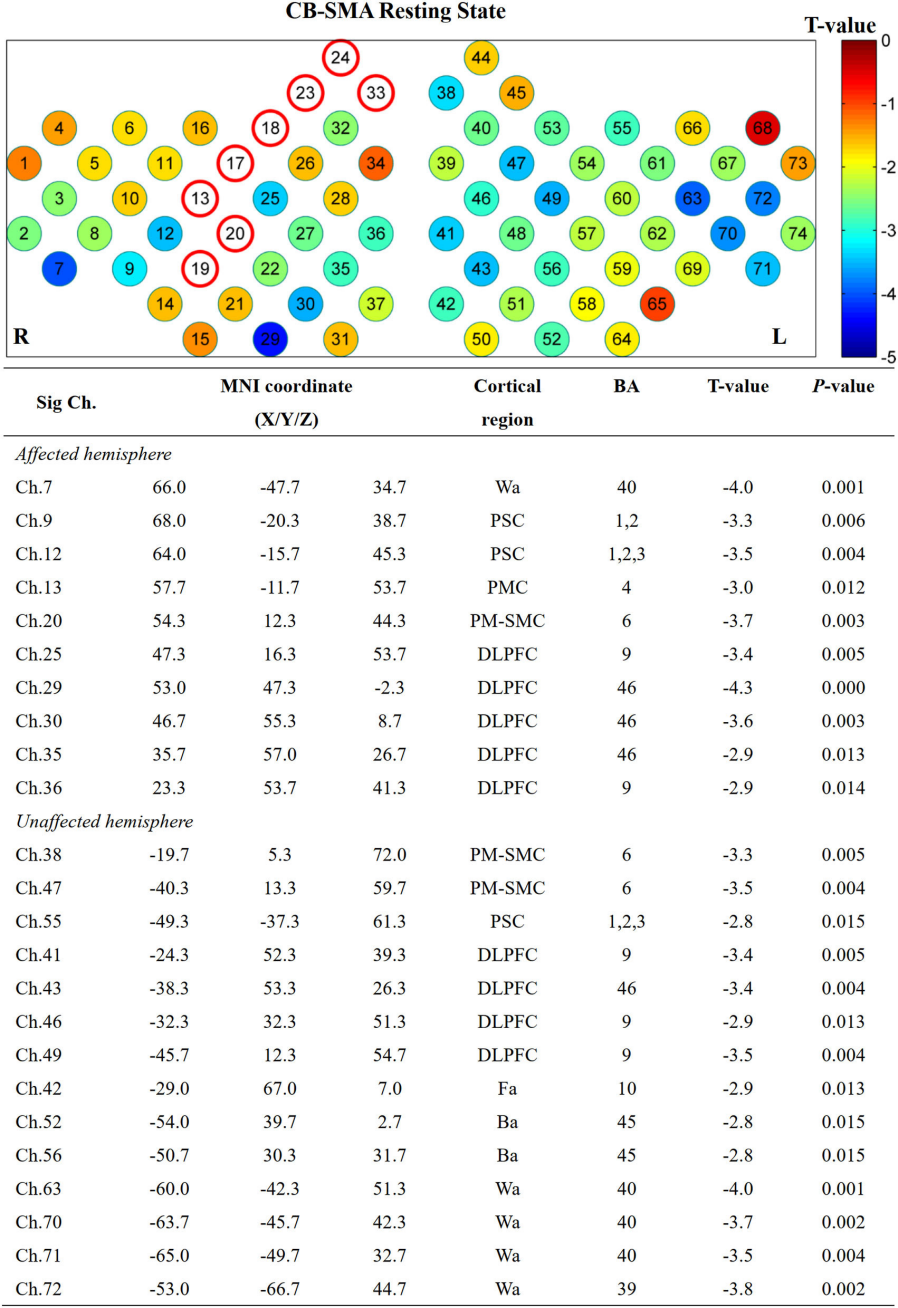
Significant channels after iTBS intervention with their MNI coordinate, Brodmann's label, *T*- and *P-*value. Wa, Wernicke's area; Ba, Broca's area; PSC, Primary somatosensory cortex; PMC, Primary motor cortex; PMSMC, Pre-Motor and supplementary motor cortex; DLPFC, Dorsolateral prefrontal cortex; Fa, Frontopolar area.

**Figure 6:**
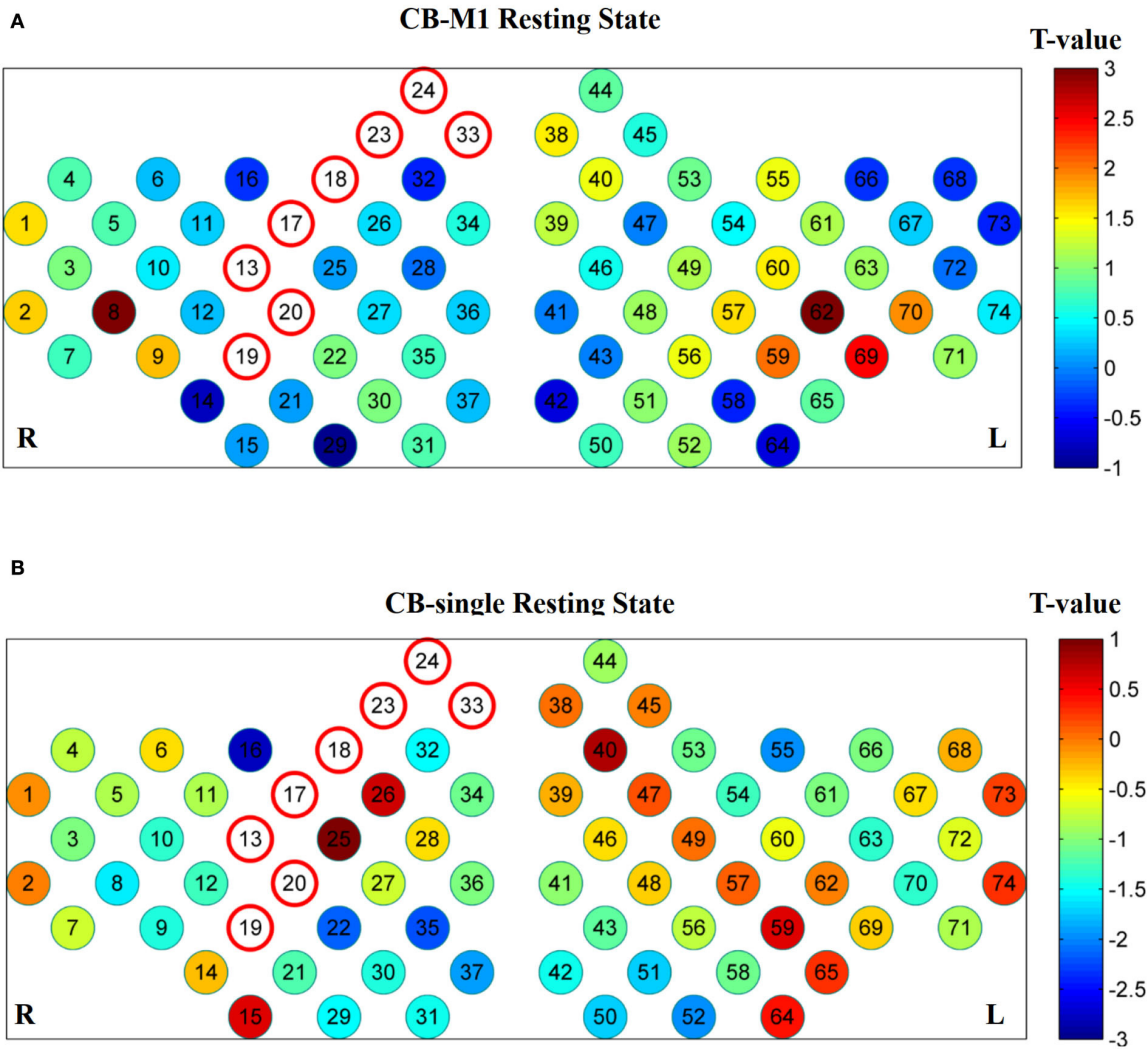
No significant connections exist after FDR correction in CB-M1 group **(A)** and CB-single group **(B)**.

Furthermore, the affected hemisphere motor network was chosen as an ROI area to calculate FC associated with the other channels. Consistent with the FC matrix model, a significant difference was observed in the CB-SMA group after the iTBS intervention. Moreover, 24 channels had meaningful variance after FDR correction (*P* < 0.0157, Figure 5), indicating the strong inhibition effect of the CB-SMA iTBS protocol. In the right hemisphere, the FC between ROI and DLPFC showed a significant difference after iTBS intervention. The strongest inhibitive effect was observed between the ROI and DLPFC representative area, as well as a within-hemisphere connection (Ch. 29, *T* = −4.3, *P* < 0.000). Inhibitive connections between the ROI and Wa (Ch. 7, *T* = −4.0, *P* = 0.001), primary somatosensory cortex (Ch. 9, *T* = −3.3, *P* = 0.006; Ch. 12, *T* = −3.5, *P* = 0.004), PMC (Ch. 13, *T* = −3.0, *P* = 0.012), and PM-SMC (Ch. 20, *T* = −3.7, *P* = 0.003) were also observed in the affected hemisphere. Meaningful inter-hemisphere connectivity existed primarily between the ROI and contralateral PM-SMC (Ch. 38, *T* = −3.3, *P* = 0.005; Ch. 47, *T* = −3.5, *P* = 0.004), DLPFC (Ch. 41, *T* = −3.4, *P* = 0.005; Ch. 43, *T* = −3.4, *P* = 0.004; Ch. 46, *T* = −2.9, *P* = 0.013; Ch. 49, *T* = −3.5, *P* = 0.004), Ba (Ch. 52, *T* = −2.8, *P* = 0.015; Ch. 56, *T* = −2.8, *P* = 0.015), and Wa (Ch. 63, *T* = −4.0, *P* = 0.001; Ch. 70, *T* = −3.7, *P* = 0.002; Ch. 71, *T* = −3.5, *P* = 0.004; Ch. 72, *T* = −3.8, *P* = 0.002). The most significant inter-hemisphere FC difference existed between the ROI and Wa (Ch. 63, *T* = −4.0, *P* = 0.001). However, there was no meaningful difference after FDR correction in the CB-M1 and CB-single target groups (Figure 6).

### Task-related activation

**Figure 7:**
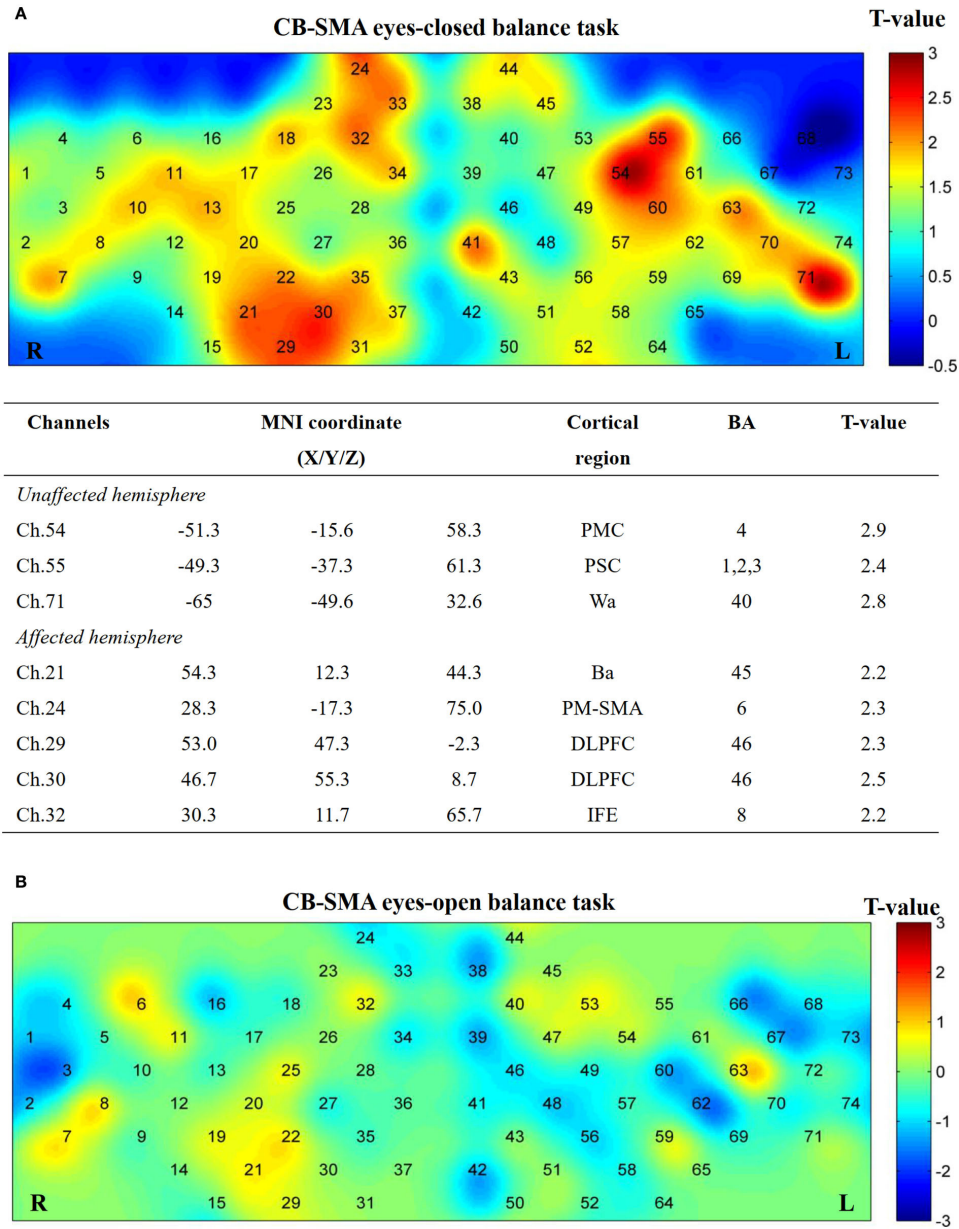
Cortical activation map in CB-SMA group in EC condition **(A)** and EO condition **(B)**. Results from paired *t*-test of beta value between post- and pre-CB-SMA iTBS protocol. None channels passed FDR correction. Channels with a meaningful trend have been listed.

No significant differences (*P* > 0.05) were observed after FDR correction among the three groups in both EO and EC conditions. However, several channels showed activation in the CB-SMA EC condition (Figure 7). On one side, the prefrontal and PMC showed important activation on the affected hemisphere, with the DLPFC area (Ch. 30, *P* = 0.03) as the most outstanding. On the other side, PMC, primary somatosensory cortex, and Wa on the unaffected hemisphere were activated during the standing task, wherein PMC (Ch. 54, *P* = 0.01) exhibited the strongest activation.

**Figure 8:**
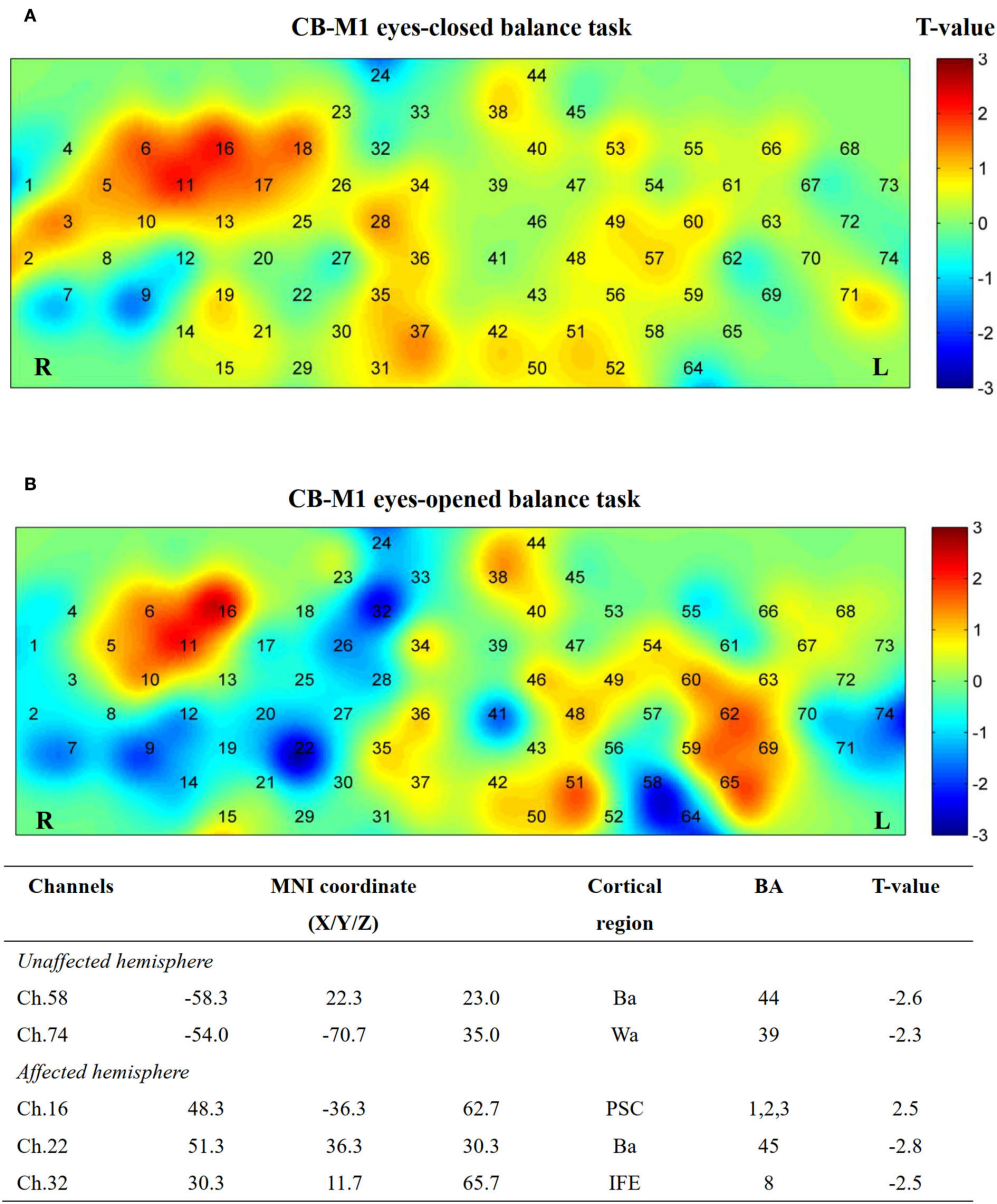
Cortical activation map in CB-M1 group in EC condition **(A)** and EO condition **(B)**. Results from paired *t*-test of beta value between post- and pre-CB-M1 iTBS protocol. None channels passed FDR correction. Channels with a meaningful trend have been listed.

Additionally, five channels showed activation differences after iTBS when conducting the EO balance task in the CB-M1 group (Figure 8). An inhibitive effect was observed on bilateral Ba (Ch. 22, *P* = 0.02; Ch. 58, *P* = 0.03).

**Figure 9:**
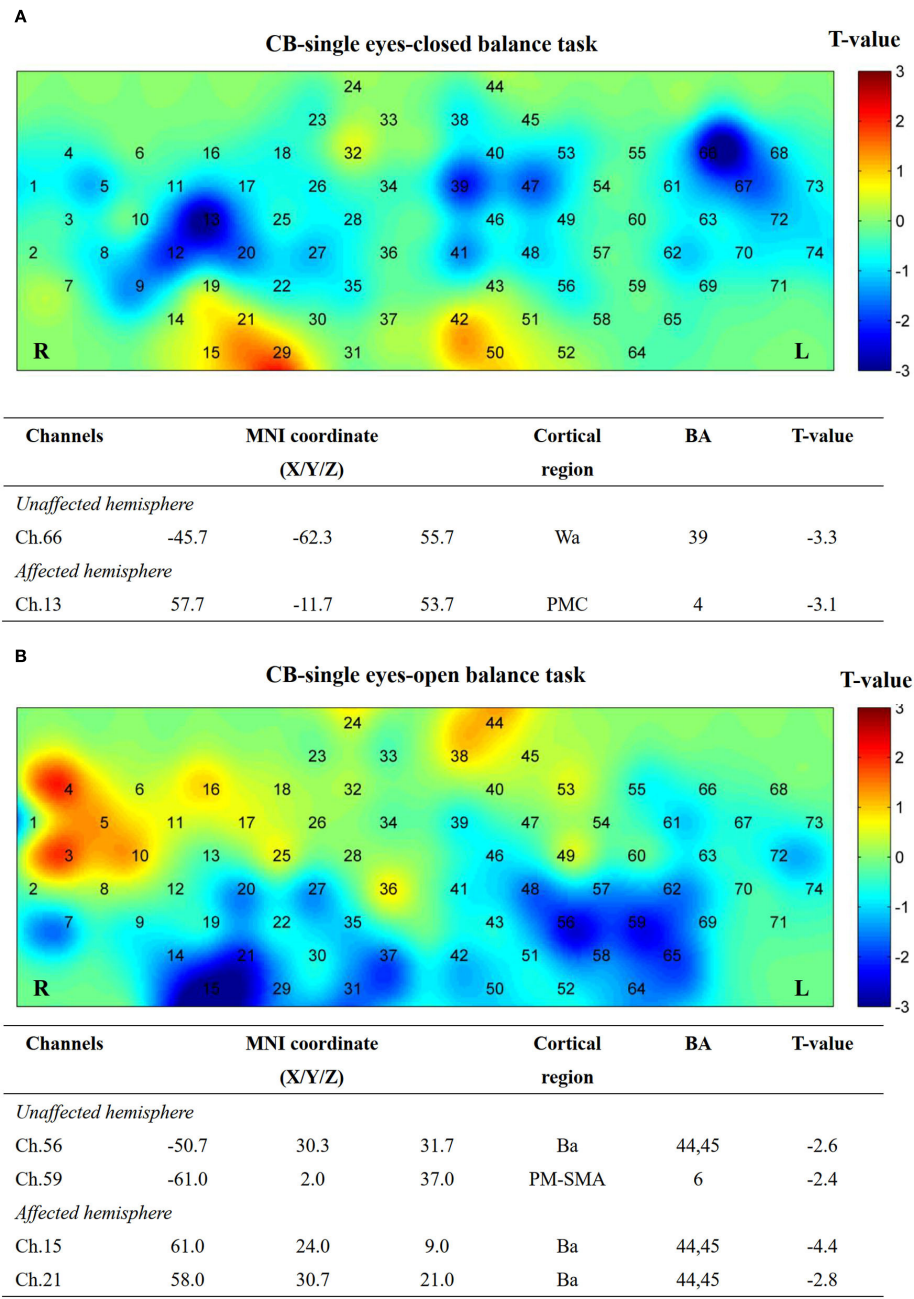
Cortical activation map in CB-single group in EC condition **(A)** and EO condition **(B)**. Results from paired *t*-test of beta value between post and pre CB-M1 iTBS protocol. None channels passed FDR correction. Channels with a meaningful trend have been listed.

After single-cerebellum iTBS stimulation, the right Wa (Ch. 66) and left PMC areas (Ch. 13) were inhibited strongly during the EC task. Furthermore, the Ba (Ch. 56, 15, and 21) and PM-SMA areas (Ch. 59) were inhibited during the EO task (Figure 9).

## Discussion

To the best of our knowledge, this study is the first to explore the potential effects of paired stimulation targets on the balance function rehabilitation of patients with stroke. Specifically, this study explored the iTBS effects of the cerebellum–cerebrum paired targets including the CB-M1, CB-SMA, and CB-single targets. We found that paired target stimulation could improve the balance function and change the cerebral connection models of patients with stroke. We found that the most significant immediate modulation effect on COP measurement is induced by CB-SMA and CB-M1 iTBS protocols under EO and EC conditions. Additionally, we also found a significant inhibition model in the CB-SMA group, covering the frontal and partial parietal lobes, which corresponds to our hypotheses.

### Changes in the center of pressure

An improvement in EC balance performance was observed in most COP parameters in the CB-SMA group, in contrast to the little change between the pre- and post-CB-M1 and CB-single iTBS intervention. Fiber associate cerebral cortex with cerebellar nuclei may be activated through the two-way facilitation effects. The nuclei in the brainstem mediate the sensorimotor information flow along the CTC pathway including the red nucleus and olive (15, 41), resulting in a better balance of somatosensory feedback and faster process speed to adjust body posture when vision was shut down. Compared with the CB-single group, the stimulation over the SMA additionally encoded the internal information process (42) and we speculate that iTBS may help with this function considering its structure fibers correlated closely (43). In another EEG research, Goel et al. (44) applied continuous TBS (cTBS) protocols over SMA and observed a smaller COP sway degree than the sham control group in online balance measurement. They thought that cTBS disrupts the connection from the SMA to other prefrontal brain areas such as the cingulate gyrus, posterior parietal cortex, and anterior cingulate, hence improving posture stability by decreasing mobility. The CB-SMA paired target stimulation in this study may also work in a similar mechanism which will be discussed later.

Group effects were also observed in the EO condition after CB-M1 target stimulation. The corticospinal excitability of M1 (45) and excitability of the peroneus longus (46) were proportional to COP velocity, especially the ML-sway control. In our results, only ML-COP_*a*_ showed improvement which is less than the changes in the AP direction. One possible explanation may be that our current targets may not raise enough neural activation of the peroneus longus and corticospinal excitability. However, the decreasing AP direction sway may help improve balance adjustment in all directions, avoiding falling risks (46).

Any immediate improvement effect was not observed, as detected by CB-single stimulation. In our previous review (47), we also did not find any convincing evidence of immediate balance improvements after a one-cerebellum iTBS session. The balance improvement effect may rely on the accumulation of the TMS effect rather than immediate changes (7, 8).

### Resting-state functional connection of brain network

Reorganization of the brain network after TMS is a crucial step in rehabilitation (28). In our resting-state analysis, we observed a significant inhibition originating from the right motor network to the contralateral motor network, and bilateral dorsal lateral and prefrontal cortex after CB-SMA iTBS. In the ROI seed analysis, we observed a significant increase in inhibition from the ROI to the bilateral DLPFC, language, motor, and sensory networks between pre- and post-CB-SMA iTBS protocols. The SMA and cerebellum communicate through the cortico-ponto-cerebellar tract and other possible CTC tracts (48, 49). The inhibition effect (50), which originated from the SMA to the cortex including the anterior cingulate cortex, angular gyrus, and middle temporal, was observed through the fMRI after a 1-Hz repetitive TMS (rTMS) on SMA, interrupting the hyper-connections from the SMA to the cerebellum. Interestingly, 10-Hz high-frequency rTMS over the SMA can also normalize brain connectivity and improve gait (51). Although the TMS protocols varied, the brain network model may indicate a reset of whole-brain FC and spare more neural resources for necessary activity instead of meaningless consumption (52, 53). One concept which may explain this decrease is that compensative excitability from the unaffected hemisphere was restored to a relatively balanced inter-hemisphere state after rehabilitation interventions. Another research (54) proved that higher baseline ipsilateral SMA-M1 (R_Motor) interconnections correlated with worse motor function recovery. Thus, our modulation effect may reverse this trend. Indeed, high within-hemisphere FC may be responsible for motor function asymmetry (55). A reset of the unregular brain network may contribute to a restoration of better motor outcomes. However, after a period of rehabilitation, Arun et al. (53) believed that a decline of resting-state FC in the contralesional hemisphere motor area, accompanied by increasing connections in the ipsilesional premotor and contralesional motor areas, represents a recovery of the normal state. We believe that this recovery effect resulted from the accumulation effect of long-time treatment (54).

In patients with essential tremors, researchers found that five-session cerebellum rTMS could restore FC in the CTC network (56). Similarly, Ma et al. (57) found that FC declined in patients with stroke after cerebellum electrical stimulation. In our immediate measurement, we found some connection declined in the CB-single group, highlighting three inter-hemisphere connections, which may be the region where the most inhibited connection resulted from the activation of the cerebellum. Halko et al. (58) found that default network connectivity, as measured by fMRI, was increased after cerebellum iTBS, as well as little changes in the motor network. Rastogi et al. (59) confirmed that cTBS on the cerebellar hemisphere could decrease FC in the cognitive network through the DTC pathway. The released excitatory of Purkinje cells may induce large inhibition on the cerebrum. However, the exact nature of the TBS effect induced in the brain cortex from the cerebellum is still uncertain (12), and a problem with different TBS protocols which have similar effects.

In the CB-M1 group, we highlighted one weak decrease in the right motor network. Both 1- and 10-Hz rTMS on M1 possess a modified effect on the motor network of patients with stroke, where high-frequency rTMS increased FC both within the and inter-hemispheres (60). We observed some potentially increased inter-hemisphere connections after CB-M1 stimulation. Necessary adjustments of TMS protocols should be applied to recreate this network model.

Finally, we did not find any significant difference between ROI and other networks after CB-M1 and CB-single intervention.

### Balance task brain activation

In the block-design balance tasks measured by fNIRS, a major activation trend (P > 0.05 FDR-corrected) was observed on ipsilateral DLPFC, PM-SMC, and contralateral PMC after CB-SMA iTBS protocol in the EC balance block-designed fNIRS task. The activation of bilateral SMA after single-CB iTBS has been observed when healthy people perform balance tasks (22). Meanwhile, the activation of the DLPFC may represent a compensation effect for demanding balance tasks when patients have sensorimotor deficits (24), as well as a representation of better neural efficiency (25).

Additionally, a target effect was observed on the group difference in the CB-M1 group compared with that in the other two groups on EO measurement. There is an inhibition trend (*P* > 0.05 FDR-corrected) in the bilateral Broca area and right hemisphere, Wernicke area, after CB-M1 iTBS protocol in EO condition. The Broca and Wernicke parts were reported to have FC with the motor area and right cerebellum in auditory speech tasks (61); however, their roles in maintaining body balance are still unknown.

Finally, an inhibition trend after single-cerebellum stimulation was witnessed in both EO and EC tasks, which may be consistent with the well-known cerebellar brain inhibition effect.

### Safety and prospect

Intermittent theta burst stimulation has been considered a safe NBS method; moreover, no adverse events, as well as the intolerance of fNIRS measurement, occurred in all patients (62, 63).

The set of paired stimulation targets based on neural communication passes also corresponds to the concept of neural circuit magnetic stimulation, which has the potential of enlarging the normal TMS effect and leading to enhanced rehabilitation (64). Neural circuits represented by CTC, especially DTC, are able to communicate, which could be utilized in enhanced rehabilitation. In this study, we explored the proper combinations of neural circuit targets for balance function promotion and possible neural network models. Our results may indicate that CB-SMA is a meaningful target pair compared with the CB-M1 and CB-single groups. Nevertheless, more stimulation pairs may also have the potential if further explored due to the neuroplasticity induced by TBS protocols.

### Limitation

This study has some limitations. First, we did not pair stimulation pulses in all three groups. Major articles researching balance rehabilitation of patients with stroke discussed the effects of 600 pulses on the cerebellum. We were not supposed to double the doses because of a lack of evidence. Moreover, the potentiation or inhibitory effect of iTBS is critically correlated with pulses delivered on neural structures. The potentiation effect of iTBS may be turned to inhibitive according to pulses delivered (65). It has been reported that conventional facilitatory iTBS turned into inhibitory when a doubled dose was applied on M1 (66). For the consideration mentioned above, we only applied 600 pulses on the cerebellum. Second, the activation analysis in EO/EC tasks failed to pass FDR; thus, the sample size must be larger for further long-term intervention effect research. Third, the lesion area in our patients was the basal ganglia region, which is too deep for fNIRS to detect. In this study, we did not collect structure MRI and failed to describe the location of participants' lesions. Furthermore, we cannot rule out some spurious signals from fNIRS when channels measure over the lesion area. Fourth, the fNIRS measurement is offline with balance evaluation where the synchronization methods were restricted by mechanical reasons. We optimized our assessments (67, 68) for better cortical activation when patients with stroke performed demanding balance tasks. Fifth, we deserted some parts of the sensory cortex and spared more channels to the frontal cortex when designing our fNIRS montage, which may lose some activation information or FC changes located on the posterior parietal cortex.

## Conclusion

In this pilot study, we compared cerebellum–cerebrum paired targets (CB-SMA and CB-M1) with CB-single stimulation targets and found an immediate benefit to EO and EC balance control in paired targets. With the fNIRS measurement, we considered CB-SMA targets as having the potential in reorganizing the brain network for a more reasonable allocation of neural resources, as an explanation for better EC balance. We believe that the CB-SMA target has a promising combination and long treatment effect; however, this must be further investigated.

## Data availability statement

The raw data supporting the conclusions of this article will be made available by the authors, without undue reservation.

## Ethics statement

The studies involving human participants were reviewed and approved by the Ethics Committee for Clinical trials of Huashan Hospital affiliated to Fudan University (approval number: 2021-644). The patients/participants provided their written informed consent to participate in this study.

## Author contributions

YX, XT, and YZ designed the experiment. RH recruited patients with stroke. YX, XT, WW, ST, CL, and QZ conducted the experiments. YX and JL analyzed the data. YX and XT interpreted the data and wrote the manuscript. All authors contributed to the article and approved the submitted version.
